# Supplementary material for: Remote neuropsychological assessment of patients with neurological disorders and injuries—a study protocol for a cross-sectional case-control validation study
Source: BMJ Open. 2024 Apr 22;14(4):e080628. doi: 10.1136/bmjopen-2023-080628 (PMC11043743; doi:10.1136/bmjopen-2023-080628)
Supplement: Author's manuscript [file bmjopen-2023-080628.draft_revisions.pdf]

BMJ Open

BMJ Open is committed to open peer review. As part of this commitment we make the peer review history of every article we publish publicly available.

When an article is published we post the peer reviewers' comments and the authors' responses online. We also post the versions of the paper that were used during peer review. These are the versions that the peer review comments apply to.

The versions of the paper that follow are the versions that were submitted during the peer review process. They are not the versions of record or the final published versions. They should not be cited or distributed as the published version of this manuscript.

BMJ Open is an open access journal and the full, final, typeset and author-corrected version of record of the manuscript is available on our site with no access controls, subscription charges or pay-per-view fees (http://bmjopen.bmj.com).

If you have any questions on BMJ Open's open peer review process please email info.bmjopen@bmj.com

BMJ Open

Remote Neuropsychological Assessment of Patients With Neurological Disorders and Injuries – A study protocol for a cross-sectional case-control validation study

Journal:	*BMJ Open*
Manuscript ID	bmjopen-2023-080628
Article Type:	Protocol
Date Submitted by the Author:	06-Oct-2023
Complete List of Authors:	Berginstrom, Nils; Umeå University, Department of Community Medicine and Rehabilitation, Rehabilitation Medicine; Umeå University, Department of Psychology<br>Andersson, Linus; Umea Universitet, Department of Psychology
Keywords:	Psychometrics, Neurological injury < NEUROLOGY, Stroke < NEUROLOGY, Parkinson-s disease < NEUROLOGY, Multiple sclerosis < NEUROLOGY

SCHOLARONE™
Manuscripts

Remote Neuropsychological Assessment of Patients with Neurological Disorders and Injuries – A study protocol for a cross-sectional case-control validation study

*Berginström, Nils[1,2], Andersson, Linus[2]

1. Department of Community Medicine and Rehabilitation, Rehabilitation Medicine, Umeå University, Umeå

2. Department of Psychology, Umeå University, Umeå

*Corresponding author at:

Department of Psychology, Umeå University, 901 87 Umeå, Sweden

E-mail address: nils.berginstrom@umu.se

Tel: +46 (0)90 – 786 88 64

Word count: 2180

For peer review only

For peer review only - http://bmjopen.bmj.com/site/about/guidelines.xhtml

Abstract

Introduction: There are great potential benefits of being able to conduct neuropsychological assessments remotely, especially for hard-to-reach or less mobile patient groups. Such tools need to be equivalent to standard tests done in the clinic and also easy to use in a variety of clinical populations.

Methods and analysis: This study protocol describes a cross-sectional study aimed at validating the newly developed digitalized neuropsychological test battery Mindmore Remote in patients with neurological disorders and injuries. Diagnoses comprise traumatic brain injury, stroke, Parkinson's Disease, multiple sclerosis, brain tumor and epilepsy. Fifty patients in each patient group will be included. In addition, 50 healthy controls will be recruited. All participants will undergo both testing with Mindmore Remote at home and traditional neuropsychological assessment in a randomized order. The primary outcome is the association between tests from the Mindmore Remote battery and their equivalent traditional neuropsychological tests. Further bias between methods and differences between groups will also be investigated.

Ethics and dissemination: The study protocol has been approved by the Swedish Ethical Review Authority (2022-06230-01) and adheres to the declaration of Helsinki. All participants will be given oral and written information about the study and sign informed consent forms before entering the study. All participants are informed that they can terminate their participation in the study at any given time, without giving any explanation, and participating at the study or not will not affect their care at the clinic. Neither authors nor personnel involved in the research project are affiliated with Mindmore AB. The results from the study will be published in peer-reviewed scientific journals and presented in national and international conferences on the topic.

Study registration number ClinicalTrials.gov, NCT05819008. Release date: April 19, 2023

Keywords: Neuropsychological Assessment, Remote Consultation, Memory, Executive Functions, Attention, Processing Speed

For peer review only - http://bmjopen.bmj.com/site/about/guidelines.xhtml

Strengths and limitations of this study

- A large cross-sectional study including 300 patients with different neurological disorders and injuries, and 50 healthy controls.
- The patients are clinical patients currently being under evaluation for cognitive deficits, making the results from the study clinically generalizable.
- If the remote test battery proves to be valid, such results would have direct practical implications in clinical practice.

For peer review only

For peer review only - http://bmjopen.bmj.com/site/about/guidelines.xhtml

Introduction

Digital meetings are now a ubiquitous part of everyday life in many parts of the world, not least following the COVID-19 pandemic that necessitated widespread use of methods for online communication. These developments have the potential to benefit healthcare in situations where physical meetings are challenging. Remote healthcare solutions have always been a pertinent issue for patients from sparsely populated areas, who have a high degree of disability, or who have difficulties or lack the resources to travel.

Patients with neurological conditions are a highlighted group in this regard. Due to injuries or progressive diseases to neurons within the central nervous system, they often struggle with logistical problems, either directly or indirectly related to their physical and mental symptoms. Diagnoses include traumatic brain injury,[1,2] stroke,[3] Parkinson's disease,[4] multiple sclerosis,[5] epilepsy,[6] and brain tumors.[7] These conditions result in structural and functional disruptions to the brain and or spine, causing both physical and mental disabilities. Deficits in cognitive and executive functioning are amongst the most common symptoms in patient groups. These characteristics render neuropsychological assessment a key part both in diagnosis and rehabilitation.[8]

Traditionally, the neuropsychological examination requires a physical meeting. The psychologist records the performance of the patient using paper-pencil test and a manual stopwatch. After the testing session, the psychologist manually scores the tests and compares the outcomes to normative performance using tables from testing manuals or peer-reviewed research articles. This is a time-consuming endeavor, with high costs for both patients and the health care system.

Computerized testing have become more common in the 21st century,[9,10] including combinations of paper-pencil tests and computers or tablets.[11,12] This have reduced the time that psychologists spend scoring results from neuropsychological tests. Traditional testing nevertheless requires the patient to physically visit a clinic for the assessment. During the last few years, there have been an increasing interest in performing neuropsychological assessment remotely[13].

A core problem is that neuropsychological assessments are difficult to adopt to a digital, remote platform. Standardization are necessary components in many tests (see for example Delis et al[14] or Wechsler[15,16]), since results are compared with a normative population who have performed the tasks under the same conditions. Changing the administration procedure too much, might make the results invalid. While some traditional tests such as simple verbal tasks may be administrated through e.g., video conference without major threats to validity, other tasks such as those requiring motor responses or visual tracking are ill-suited for such solutions[17,18]. There are some examples of neuropsychological testing platforms that also includes automated administration and scoring procedures, such as Test My Brain Digital Neuropsychology Toolkit[19], Philips IntelliSpace Cognition[20] or the Cogniciti's Brain Health Assessment[21]. It is, however, necessary to thoroughly validate such tools before they can be implemented in healthcare.

Mindmore (www.mindmore.com) is an automated digital application originally distributed to patients on site at a clinic or healthcare provider using a tablet device. Its constituent tests have been validated in healthy adults[11] and include normative data scores[22]. A remote version (Mindmore Remote) is currently under development, so that patients can complete neuropsychological testing procedures at home without any health care personnel present. Mindmore Remote is currently undergoing validation, and normative data is being collected from healthy adults (van den Hurk, personal communication, approved ethics application by the Swedish Ethical Review Authority, dnr 2019-02030). However, the instrument does currently not include data from patient groups with cognitive impairments. This protocol presents a research project aiming to validate Mindmore Remote in patients with neurological disorders and injuries.

Objectives

The primary aim of this project is to assess the validity of Mindmore Remote tests by comparing them with standardized neuropsychological tests. We pose the following research questions:

1. Are the tests in Mindmore Distance equivalent to traditional neuropsychological tests in patients with traumatic brain injury, stroke, multiple sclerosis, Parkinson's Disease, epilepsy, and brain tumor?
2. Can the results from Mindmore Distance be transferred into neuropsychological profiles (e.g., the distribution of performance across several tests) that can be used in diagnostics for specific patient groups?
3. How do the patients experience undergoing a neuropsychological evaluation on their own compared to traditional neuropsychological assessment in a physical meeting with a psychologist?

Methods and analysis

Study design and setting

This study is a cross-sectional study with a case-control design. All reporting of results in peer-reviewed scientific journals will adhere to the STROBE guidelines[23]. The study is located at the Neuro-Head-Neck-Centre (NHHC), including departments of Neurology, Neurosurgery and Neurorehabilitation at Umeå University Hospital, Umeå. Data collection will be conducted during March 2023 to December 2025.

Participants

Patients with the diagnoses traumatic brain injury, stroke, Parkinson's Disease, multiple sclerosis, epilepsy, and glioma who are referred for neuropsychological assessment at NHHC will be offered to participate in the study. The aim is to include 50 patients in each diagnostic group.

Healthy adults (n = 50) will be recruited as controls through advertising at Umeå University Hospital. Since the patient groups will differ in age variance, the aim is to recruit a control group with a variance in age. The controls will be reimbursed for their participation (100 SEK; approximately 10 €).

For peer review only - http://bmjopen.bmj.com/site/about/guidelines.xhtml

Eligibility criteria

The inclusion and exclusion criteria are the same for patients and healthy controls, except that healthy controls should not be diagnosed with any neurological disorders.

Inclusion criteria

- Physician-generated neurological diagnosis of either traumatic brain injury, stroke, Parkinson's Disease, multiple sclerosis, epilepsy, and glioma.
- Age 18 or above
- In possession of a computer with internet connection

Exclusion criteria

- Any other neurological diagnosis than the primary diagnosis (for healthy controls, any neurological diagnosis).
- Severe psychiatric disorder, including schizophrenia or other psychotic disorder, severe depression, bipolar disorder, and suicidality.
- Addiction disorder, including both alcohol and other substances.
- Not speaking Swedish
- Not being able to participate in a neuropsychological examination, or to give informed consent due to physical or mental disability.

Instruments

Primary outcome measures: Neuropsychological tests

All participants will be tested using all tests in the Mindmore Remote battery, and corresponding traditional neuropsychological tests. Table 1 summarizes all tests in Mindmore Remote, and provides a detailed description of each test, primary outcome measures and the corresponding traditional neuropsychological test that will be used to test the validity of each Mindmore Remote test. The test

For peer review only - http://bmjopen.bmj.com/site/about/guidelines.xhtml

battery will be performed in the order given in Table 1, with the addition that the RAVLT Retention and Recognition trials will be performed after Stroop in the Mindmore Remote battery, and Word List Recall and Recognition Trials will be performed after Color-Word-Interference Test in the traditional battery.

Raw scores of the tests will be used as the main variables in the analyses. However, scaled scores (i.e., those computed from the normative samples in the test manuals of the traditional tests and from the built-in norms in Mindmore Remote) might also be considered in some analyses.

[Insert Table 1: Outcome measures here]

Secondary Outcome Measures: Self-Assessment questionnaires

In addition to the neuropsychological tests, all participants will also complete self-assessment questionnaires. The results from these questionnaires will mainly be used to adjust for effects of possible confounders when analyzing neuropsychological test data. The following self-assessment questionnaires will be used: Hospital Anxiety and Depression Scale (Zigmond & Snaith, 1983); Insomnia Severity Index (Bastien, Vallieres, & Morin, 2001); Cognitive Failure Questionnaire (Broadbent, Cooper, FitzGerald, & Parkes, 1982); Perceived Stress Scale (Levenstein et al., 1993); and Multidimensional Fatigue Inventory (Smets, Garssen, Bonke, & De Haes, 1995). Participants will also answer background questions regarding age, education, gender identity, occupation, number of hours slept last night, and medication, including medication during the last 24 hours.

Data Collection and Management

Assessment Procedure

After reading and signing informed consent forms, patients will be randomized into one of two test sequences – either beginning with performing the Mindmore Remote tests at home, followed by traditional neuropsychological testing on site at the clinic, or vice versa (Figure 1). In cases where the

For peer review only

For peer review only - http://bmjopen.bmj.com/site/about/guidelines.xhtml

data collection is a part of a larger neuropsychological examination, the validation battery will be performed first during that examination, to avoid effects of fatigue. The test will be administered in the order outlined in table 1. Healthy controls will be randomized in a similar manner as patients, and except that data collection might be a part of a larger neuropsychological examination for patients, the data collection will not differ between groups.

All data collection will be performed by psychologists trained specifically for the task of neuropsychological examinations. The two test sessions will be performed within two weeks, with at least one day of resting in between. Each test session will last about 30 minutes, adding up to one hour of testing time for both assessments in total. In adjacent to the traditional neuropsychological examination, patients will complete self-assessment questionnaires. After data collection, a subsample of 10 patients will be contacted and offered to participate in a telephone interview regarding research question 3.

[Insert Figure 1. Flow-chart of the study here]

Sample Size

To be able to show that one patient group has a different cognitive profile compared to other patients or healthy controls, the aim is to include 50 participants in each group, which is the recommendation for sample size in normative studies of neuropsychological tests[24]. With alpha = .05 and a power of 80%, 16 participants would be enough to detect differences of one standard deviation from the mean. With the goal of 50 participants in each group, this means a statistical power of 0.999 to detect differences of one standard deviation from the mean, and a statistical power of 0.706 to detect differences of 0.5 standard deviations from the mean.

Data Analysis Plan

For peer review only

For peer review only - http://bmjopen.bmj.com/site/about/guidelines.xhtml

Validation of Mindmore test against traditional paper-pencil neuropsychological test will be performed using Pearson and/or Spearman correlation coefficients depending on distribution of data. To evaluate bias between methods, the Bland-Altman method[25] will be used. In addition, multiple linear regressions will be performed to investigate and adjust for the influence of background variables, such as age, education and gender. To establish neuropsychological profiles of each diagnostic group, analysis of variance, with and without covariates, will be performed to determine differences between groups.

Patient and public involvement

Local patient organizations have been informed about the research project, and results from the study will be presented at meetings of these organizations.

Ethics and Dissemination

The study protocol has been approved by the Swedish Ethical Review Authority (2022-06230-01). Before initiating any form of neuropsychological assessment, all participants will be given oral and written information about the study and their participation and will sign informed consent. All participants are informed that they can abort their participation at any given time, without having to give reasons for this. For patients, participating in the study or not will not affect regular health care given at the clinic.

Undergoing a neuropsychological examination (remotely or traditionally) does not come with any risks in itself. However, it might be stressful and anxiety-provoking, especially for participants getting the impression that they are not performing well. This is a common clinical procedure, and the test leaders, all clinical psychologists, are us to handle such reactions during test sessions.

Data management

For peer review only - http://bmjopen.bmj.com/site/about/guidelines.xhtml

After inclusion, all participants will be assigned a code. All study material will be coded, and a code key is stored separately in a secure compartment, only accessible to researchers within the project. Only personnel within the research project will have access to data. Study data will be stored for at least ten years after completion of the study.

For Mindmore Remote, the data storage is sophisticated and secure, and no personal data is stored on Mindmore's servers. The invitation to the test is sent by a pseudonymized link, and only the researchers within the project have access to the code, as stated above. In this way, Mindmore AB cannot identify any person taking the test, although the test results are stored on their server.

Plans for communicating important protocol amendments to relevant parties

In case the study protocol will undergo modifications or amendments, an additional ethical application will be submitted to the Swedish Ethical Review Authority. Accordingly, changes will be made in the trial registry (Clinicaltrials.gov).

Dissemination

Results from the study will be published in peer-reviewed open access scientific journals. Further, results will also be presented at national and international scientific conferences, as well as shared with patient organizations and health care officials and policy makers.

Authors' contributions

NB is the principal investigator and responsible for the project, and for the reporting of results from the project. Data collection will be performed by clinical neuropsychologists, at Neuro-Head-Neck Centre, Umeå University hospital, all under the supervision of the principal investigator. NB wrote the initial draft of the manuscript. LA contributed to the study design and revised the manuscript for intellectual content.

Funding

For peer review only - http://bmjopen.bmj.com/site/about/guidelines.xhtml

The study is supported by the Research and Development Fund granted by the County Council of Västerbotten [RV-978915], Stiftelsen Promobilia [A23004], the Swedish Neuropsychological Society and a memorial gift to the County Council of Västerbotten regarding research at Umeå University Hospital.

Competing interests

None declared.

Patient consent for publication

Not applicable.

Acknowledgements

The authors would like to thank Malin Nygård, Sofia Näckter, Emma Wärn, Felix Sjögren and Therese Eriksson for assisting in data collection.

For peer review only

For peer review only - http://bmjopen.bmj.com/site/about/guidelines.xhtml

References

1. Dikmen SS, Machamer JE, Winn HR, Temkin NR. Neuropsychological Outcome at 1-Year Post Head-Injury. *Neuropsychology*. Jan 1995;9(1):80-90. doi:Doi 10.1037/0894-4105.9.1.80
2. van der Naalt J, van Zomeren AH, Sluiter WJ, Minderhoud JM. One year outcome in mild to moderate head injury: the predictive value of acute injury characteristics related to complaints and return to work. *J Neurol Neurosurg Psychiatry*. Feb 1999;66(2):207-13.
3. Sun JH, Tan L, Yu JT. Post-stroke cognitive impairment: epidemiology, mechanisms and management. *Ann Transl Med*. Aug 2014;2(8):80. doi:10.3978/j.issn.2305-5839.2014.08.05
4. Yang Y, Tang BS, Guo JF. Parkinson's Disease and Cognitive Impairment. *Parkinsons Dis*. 2016;2016:6734678. doi:10.1155/2016/6734678
5. Benedict RHB, Amato MP, DeLuca J, Geurts JJG. Cognitive impairment in multiple sclerosis: clinical management, MRI, and therapeutic avenues. *Lancet Neurol*. Oct 2020;19(10):860-871. doi:10.1016/S1474-4422(20)30277-5
6. Holmes GL. Cognitive impairment in epilepsy: the role of network abnormalities. *Epileptic Disord*. Jun 2015;17(2):101-16. doi:10.1684/epd.2015.0739
7. Pendergrass JC, Targum SD, Harrison JE. Cognitive Impairment Associated with Cancer: A Brief Review. *Innov Clin Neurosci*. Feb 1 2018;15(1-2):36-44.
8. Lezak MD, Howieson DB, Loring DW. *Neuropsychological Assessment*. 4 ed. Oxford University Press; 2004.
9. Buckley RF, Sparks KP, Papp KV, et al. Computerized Cognitive Testing for Use in Clinical Trials: A Comparison of the NIH Toolbox and Cogstate C3 Batteries. *J Prev Alzheimers Dis*. 2017;4(1):3-11. doi:10.14283/jpad.2017.1
10. Germine L, Reinecke K, Chaytor NS. Digital neuropsychology: Challenges and opportunities at the intersection of science and software. *Clin Neuropsychol*. Feb 2019;33(2):271-286. doi:10.1080/13854046.2018.1535662
11. Bjorngrim S, van den Hurk W, Betancort M, Machado A, Lindau M. Comparing Traditional and Digitized Cognitive Tests Used in Standard Clinical Evaluation - A Study of the Digital Application Minnemera. *Front Psychol*. 2019;10:2327. doi:10.3389/fpsyg.2019.02327
12. Daniel MH, Wahlstrom D, Zhang O. *Equivalence of Q-interactive® and Paper Administrations of Cognitive Tasks: WISC®–V*. NCS Pearson Inc.; 2014.
13. Van Patten R. Introduction to the Special Issue - Neuropsychology from a distance: Psychometric properties and clinical utility of remote neurocognitive tests. *J Clin Exp Neuropsychol*. Oct 2021;43(8):767-773. doi:10.1080/13803395.2021.2021645
14. Delis DC, Kaplan E, Kramer JH. *D-KEFS - Delis Kaplan Executive Functions System – Technical Manual*. Pearson; 2001.
15. Wechsler D. *Wechsler Memory Scale - Third Edition*. Psychological Corporation; 1997.
16. Wechsler D. *Wechsler Adult Intelligence Scale—Fourth Edition*. Pearson; 2008.
17. Brearly TW, Shura RD, Martindale SL, et al. Neuropsychological Test Administration by Videoconference: A Systematic Review and Meta-Analysis. *Neuropsychol Rev*. Jun 2017;27(2):174-186. doi:10.1007/s11065-017-9349-1
18. Marra DE, Hamlet KM, Bauer RM, Bowers D. Validity of teleneuropsychology for older adults in response to COVID-19: A systematic and critical review. *Clin Neuropsychol*. Oct - Nov 2020;34(7-8):1411-1452. doi:10.1080/13854046.2020.1769192
19. Singh S, Strong RW, Jung L, et al. The TestMyBrain Digital Neuropsychology Toolkit: Development and Psychometric Characteristics. *J Clin Exp Neuropsychol*. Oct 2021;43(8):786-795. doi:10.1080/13803395.2021.2002269
20. Vermeent S, Spaltman M, van Elswijk G, Miller JB, Schmand B. Philips IntelliSpace Cognition digital test battery: Equivalence and measurement invariance compared to traditional analog test versions. *Clin Neuropsychol*. Nov 2022;36(8):2278-2299. doi:10.1080/13854046.2021.1974565
21. Paterson TSE, Sivajohan B, Gardner S, et al. Accuracy of a Self-Administered Online Cognitive Assessment in Detecting Amnestic Mild Cognitive Impairment. *J Gerontol B Psychol Sci Soc Sci*. Feb 3 2022;77(2):341-350. doi:10.1093/geronb/gbab097
22. Van den Hurk W, Bergman I, Machado A, Bjermo J, Gustavsson A. Swedish Normative Data for Mindmore: A Comprehensive Cognitive Screening Battery, Both Digital and Self-Administrated. *J Int Neuropsych Soc*. Feb 2022;28(2):188-202. doi:Pii S135561772100045x10.1017/S135561772100045x

For peer review only - http://bmjopen.bmj.com/site/about/guidelines.xhtml

23. von Elm E, Altman DG, Egger M, Pocock SJ, Gøtzsche PC, Vandenbroucke JP. The Strengthening the Reporting of Observational Studies in Epidemiology (STROBE) statement: guidelines for reporting observational studies. *Ann Intern Med*. Oct 16 2007;147(8):573-7. doi:10.7326/0003-4819-147-8-200710160-00010

24. Mitrushina MN, Boone KB, D'Elia LF. *Handbook of Normative Data for Neuropsychological Assessment*. Oxford University Press; 1999.

25. Giavarina D. Understanding Bland Altman analysis. *Biochem Med (Zagreb)*. 2015;25(2):141-51. doi:10.11613/BM.2015.015

For peer review only

Tables

Table 1: Outcome Measures

Mindmore Distance Test	Description	Main Outcomes	Validity Test
Symbol Digit Processing Test	SDPT is a test of attention and mental processing speed. The participant views a digit-symbol code key on the top of the screen. In the middle of the screen, one symbol appears. The task is to press the correct number a quickly as possible using the mouse on a number pad at the bottom of the screen.	Number of correct items coded over 90 seconds.	Coding from WAIS-IV (Wechsler, 2010)
Rey Auditory Verbal Learning Test	RAVLT is a test of verbal learning and episodic memory. The participant will hear 15 words presented over five trials, with a recall task after each trial. After the learning trial, a distraction list of 15 words is administered, and after this the task is to once again recall the first list. After 30 minutes (after Stroop below), a free recall retention trial i performed, and after this, a recognition trial, where the participant is supposed to identify the words of the first list in a list of 15 hits and 15 distractors. All responses are given verbally.	Total Learning - Number of words recalled during all five trials; Short term Recall - Number of words recalled after the distraction list; Long term recall - Number of words recalled after 30 minutes; Recognition - Number of correct hits and true negatives during recognition	Word List Recall I and II from WMS-III (Wechsler, 1997)
Corsi Block	Corsi Block is a test of short-term and working memory. On the screen, ten white squares will appear. These will blink in yellow in specific sequences of increasing length, starting with two items. Two different sequences will be presented of each length. After each sequence has finished, the participant is supposed to click with the mouse on the squares in the same order (Corsi Forwards), or in reverse order (Corsi Backwards). If the participant fails to produce the correct sequence in both trials on the same level, the test will end. Corsi Forwards is performed first, and then Corsi Backwards.	Corsi Block Forwards - Number of correct trials in the forward condition; Corsi Block Backwards - Number of correct trials in the Backward condition;	Block Span from WMS-III (Wechsler, 1997)

For peer review only

For peer review only - http://bmjopen.bmj.com/site/about/guidelines.xhtml

Trail Making Test - Click	TMT is a test of attention, visuomotor speed and the executive function of switching. The test consists of three conditions. In condition 1 (Number Sequencing), the participant will see numbers 1 to 25 in small circles on the screen and is supposed to click on the numbers in the correct order. In condition 2 (Switching), both numbers 1-13 and letters A-M appears in the circles. The participant is supposed to click on the letters and numbers in order, but each number and letter interchangeably - 1-A-2-B-3-etc. In condition 3 (Motor Speed), the circles are connected by a dotted line, and the participant is instructed to click on the circle in the same order as the line connects them.	Time to completion of each trial	Trail Making Test from D-KEFS (Delis et al., 2001)
Stroop	Stroop is a test of the executive function inhibition, performed in two conditions. In condition 1 (Color Naming and Reading) a color word appear, printed in the same color. The participant is instructed to click as quickly as possible on the correct color word (blue, red, green or yellow), spelled out in black color below the target word. In condition 2 (Inhibition) the color words are written in a different color. The participant is instructed to click, as quickly as possible, on the color word written in black color below the target word that matches the color of the word, and not the word in itself.	Time to completion of each trial	Color-Word Interference Test from D-KEFS (Delis et al., 2001)
FAS	In FAS, a test of verbal production and executive functions, the participant is instructed to say as many words as possible during one minute, starting with a specific letter. The test is performed in three trials using letters F, A, and S, respectively.	Number of words produced over all trials.	Verbal Fluency from D-KEFS (Delis et al., 2001)

Note: WAIS = Wechsler Adult Intelligence Scale; WMS = Wechsler Memory Scale; D-KEFS = Delis-Kaplan Executive Functions System. All tests will be performed using Swedish versions

For peer review only - http://bmjopen.bmj.com/site/about/guidelines.xhtml

Figure caption

Figure 1. Flow-chart of the study.

Note: After signing informed consent (1), participants will be randomized (2) into performning traditional neuropsychological examination (NPS) at the clinic first and then Mindmore remote at home or vice versa (3).

For peer review only

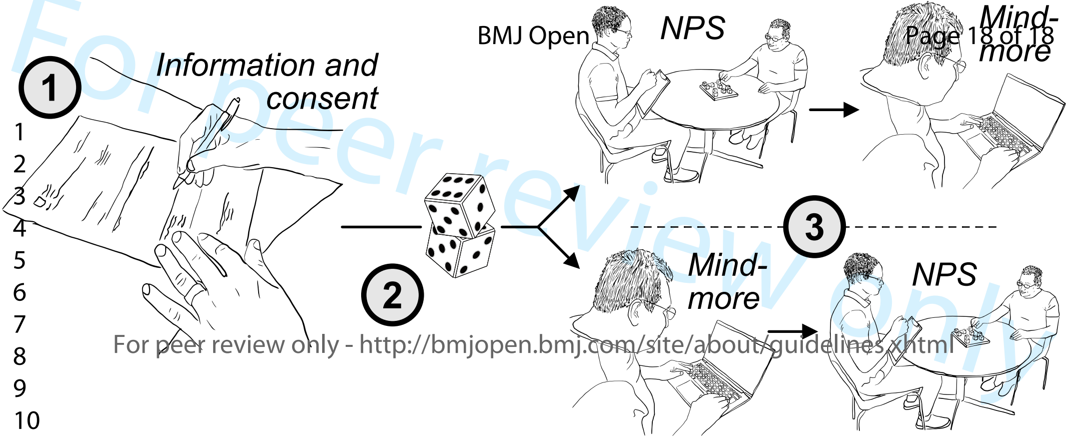

For peer review only - http://bmjopen.bmj.com/site/about/guidelines.xhtml

BMJ Open

Remote Neuropsychological Assessment of Patients With Neurological Disorders and Injuries – A study protocol for a cross-sectional case-control validation study

Journal:	*BMJ Open*
Manuscript ID	bmjopen-2023-080628.R1
Article Type:	Protocol
Date Submitted by the Author:	19-Mar-2024
Complete List of Authors:	Berginstrom, Nils; Umeå University, Department of Community Medicine and Rehabilitation, Rehabilitation Medicine; Umeå University, Department of Psychology<br>Andersson, Linus; Umea Universitet, Department of Psychology
**Primary Subject Heading**:	Neurology
Secondary Subject Heading:	Neurology
Keywords:	Psychometrics, Neurological injury < NEUROLOGY, Stroke < NEUROLOGY, Parkinson-s disease < NEUROLOGY, Multiple sclerosis < NEUROLOGY

SCHOLARONE™
Manuscripts

Remote Neuropsychological Assessment of Patients with Neurological Disorders and Injuries – A study protocol for a cross-sectional case-control validation study

*Berginström, Nils[1,2], Andersson, Linus[2]

1. Department of Community Medicine and Rehabilitation, Rehabilitation Medicine, Umeå University, Umeå

2. Department of Psychology, Umeå University, Umeå

*Corresponding author at:

Department of Psychology, Umeå University, 901 87 Umeå, Sweden

E-mail address: nils.berginstrom@umu.se

Tel: +46 (0)90 – 786 88 64

Word count: 2346

For peer review only

Abstract

Introduction: There are great potential benefits of being able to conduct neuropsychological assessments remotely, especially for hard-to-reach or less mobile patient groups. Such tools need to be equivalent to standard tests done in the clinic and also easy to use in a variety of clinical populations.

Methods and analysis: This study protocol describes a cross-sectional study aimed at validating the newly developed digitalized neuropsychological test battery Mindmore Remote in patients with neurological disorders and injuries. Diagnoses comprise traumatic brain injury, stroke, Parkinson's Disease, multiple sclerosis, brain tumor and epilepsy. Fifty patients in each patient group will be included. In addition, 50 healthy controls will be recruited. All participants will undergo both testing with Mindmore Remote at home and traditional neuropsychological assessment face-to-face in a randomized order. The primary outcome is the association between tests from the Mindmore Remote battery and their equivalent traditional neuropsychological tests. Further bias between methods and differences between groups will also be investigated.

Ethics and dissemination: The study protocol has been approved by the Swedish Ethical Review Authority (2022-06230-01) and adheres to the declaration of Helsinki. All participants will be given oral and written information about the study and sign informed consent forms before entering the study. All participants are informed that they can terminate their participation in the study at any given time, without giving any explanation, and participating at the study or not will not affect their care at the clinic. Neither authors nor personnel involved in the research project are affiliated with Mindmore AB. The results from the study will be published in peer-reviewed scientific journals and presented in national and international conferences on the topic.

Study registration number ClinicalTrials.gov, NCT05819008. Release date: April 19, 2023

Keywords: Neuropsychological Assessment, Remote Consultation, Memory, Executive Functions, Attention, Processing Speed

For peer review only - http://bmjopen.bmj.com/site/about/guidelines.xhtml

Strengths and limitations of this study

- A large cross-sectional study including 300 patients with different neurological disorders and injuries, and 50 healthy controls.
- The patients are clinical patients currently being under evaluation for cognitive deficits, making the results from the study clinically generalizable.
- If the remote test battery proves to be valid, such results would have direct practical implications in clinical practice.
- Excluding participants with languages and cultural backgrounds other than Swedish limits the generalizability of the findings to the entire Swedish population

For peer review only

For peer review only - http://bmjopen.bmj.com/site/about/guidelines.xhtml

Introduction

Digital meetings are now a ubiquitous part of everyday life in many parts of the world, not least following the COVID-19 pandemic that necessitated widespread use of methods for online communication. These developments have the potential to benefit healthcare in situations where physical meetings are challenging. Remote healthcare solutions have always been a pertinent issue for patients from sparsely populated areas, who have a high degree of motor and/or cognitive disability, or who have difficulties or lack the resources to travel.

Patients with neurological conditions are a highlighted group in this regard. Due to injuries or progressive diseases within the central nervous system, they often struggle with logistical problems, either directly or indirectly related to their physical and mental symptoms. Diagnoses include traumatic brain injury,(1, 2) stroke,(3) Parkinson's disease,(4) multiple sclerosis,(5) epilepsy,(6) and brain tumors.(7) These conditions result in structural and functional disruptions to the brain and or spine, causing both physical and cognitive disabilities. Deficits in cognitive and executive functioning are amongst the most common symptoms in patient groups. These characteristics render neuropsychological assessment a key part both in diagnosis and rehabilitation.(8)

Traditionally, the neuropsychological examination requires a physical meeting. The psychologist records the performance of the patient using paper-pencil test and a manual stopwatch. After the testing session, the psychologist manually scores the tests and compares the outcomes to normative performance using tables from testing manuals or peer-reviewed research articles. This is a time-consuming endeavor, with high costs for both patients and the health care system.

Computerized testing have become more common in the 21st century,(9, 10) including combinations of paper-pencil tests and computers or tablets.(11, 12) This have reduced the time that psychologists spend scoring results from neuropsychological tests. Traditional testing nevertheless requires the patient to physically visit a clinic for the assessment. During the last few years, there have been an increasing interest in performing neuropsychological assessment remotely(13).

For peer review only - http://bmjopen.bmj.com/site/about/guidelines.xhtml

A core problem is that neuropsychological assessments are difficult to adopt to a digital, remote platform. Standardization are necessary components in many tests (see for example Delis et al(14) or Wechsler(15, 16)), since results are compared with a normative population who have performed the tasks under the same conditions. Changing the administration procedure too much, might make the results invalid. While some traditional tests such as simple verbal tasks may be administrated through e.g., video conference without major threats to validity, other tasks such as those requiring motor responses or visual tracking are ill-suited for such solutions(17, 18). There are some examples of neuropsychological testing platforms that also include automated administration and scoring procedures, such as Test My Brain Digital Neuropsychology Toolkit(19), Philips IntelliSpace Cognition(20) or the Cogniciti's Brain Health Assessment(21). It is, however, necessary to thoroughly validate such tools before they can be implemented in healthcare.

Mindmore (www.mindmore.com) is an automated digital application originally distributed to patients on site at a clinic or healthcare provider using a tablet device. Its constituent tests have been validated in healthy adults(11) and include normative data scores(22). A remote version (Mindmore Remote) is currently under development, so that patients can complete neuropsychological testing procedures at home without any health care personnel present. Mindmore Remote is currently undergoing validation, and normative data is being collected from healthy adults (van den Hurk, personal communication, approved ethics application by the Swedish Ethical Review Authority, dnr 2019-02030). However, the instrument does currently not include data from patient groups with cognitive impairments. This protocol presents a research project aiming to validate Mindmore Remote in patients with neurological disorders and injuries.

Objectives

The primary aim of this project is to assess the convergent validity of Mindmore Remote tests by comparing them with standardized neuropsychological tests. We pose the following research questions:

For peer review only - http://bmjopen.bmj.com/site/about/guidelines.xhtml

1. Are the tests in Mindmore Remote equivalent to traditional neuropsychological tests in patients with traumatic brain injury, stroke, multiple sclerosis, Parkinson's Disease, epilepsy, and brain tumor?
2. Can the results from Mindmore Remote be transferred into neuropsychological profiles (e.g., the distribution of performance across several tests) that can be used for patients with Parkinsons' Disease and Multiple Sclerosis?
3. How do the patients experience undergoing a neuropsychological evaluation on their own compared to traditional neuropsychological assessment in a physical meeting with a psychologist?

Methods and analysis

Study design and setting

This study is a cross-sectional study with a case-control design. All reporting of results in peer-reviewed scientific journals will adhere to the STROBE guidelines(23). The study is located at the Neuro-Head-Neck-Centre (NHHC), including departments of Neurology, Neurosurgery and Neurorehabilitation at Umeå University Hospital, Umeå. Data collection will be conducted during March 2023 to December 2025.

Participants

Patients with the diagnoses traumatic brain injury, stroke, Parkinson's Disease, multiple sclerosis, epilepsy, and glioma who are referred for neuropsychological assessment at NHHC will be offered to participate in the study. The aim is to include 50 patients in each diagnostic group.

Healthy adults (n = 50) will be recruited as controls through advertising at Umeå University Hospital. Since the patient groups will differ in age variance, the aim is to recruit a control group with a variance in age. The controls will be reimbursed for their participation (100 SEK; approximately 10 €).

For peer review only - http://bmjopen.bmj.com/site/about/guidelines.xhtml

Eligibility criteria

The inclusion and exclusion criteria are the same for patients and healthy controls, except that healthy controls should not be diagnosed with any neurological disorders.

Inclusion criteria

- Physician-generated neurological diagnosis of either traumatic brain injury, stroke, Parkinson's Disease, multiple sclerosis, epilepsy, and glioma.
- Age 18 or above
- In possession of a computer with internet connection

Exclusion criteria

- Any other neurological diagnosis than the primary diagnosis (for healthy controls, any neurological diagnosis).
- Severe psychiatric disorder, including schizophrenia or other psychotic disorder, severe depression, bipolar disorder, and suicidality.
- Addiction disorder, including both alcohol and other substances.
- Not speaking Swedish, have lived in Sweden less than 10 years and/or have had a majority of education outside of the Swedish educational system.
- Not being able to participate in a neuropsychological examination, or to give informed consent due to physical or mental disability.

Instruments

Primary outcome measures: Neuropsychological tests

All participants will be tested using all tests in the Mindmore Remote battery, and corresponding traditional neuropsychological tests. Table 1 summarizes all tests in Mindmore Remote, and provides a detailed description of each test, primary outcome measures and the corresponding traditional

For peer review only - http://bmjopen.bmj.com/site/about/guidelines.xhtml

neuropsychological test that will be used to test the validity of each Mindmore Remote test. The test battery will be performed in the order given in Table 1, with the addition that the RAVLT Retention and Recognition trials will be performed after Stroop in the Mindmore Remote battery, and Word List Recall and Recognition Trials will be performed after Color-Word-Interference Test in the traditional battery.

Raw scores of the tests will be used as the main variables in the analyses. However, scaled scores (i.e., those computed from the normative samples in the test manuals of the traditional tests and from the built-in norms in Mindmore Remote) might also be considered in some analyses.

[Insert Table 1: Outcome measures here]

Secondary Outcome Measures: Self-Assessment questionnaires

In addition to the neuropsychological tests, all participants will also complete paper-pencil versions of self-assessment questionnaires. The results from these questionnaires will mainly be used to adjust for effects of possible confounders when analyzing neuropsychological test data. The following self-assessment questionnaires will be used: Hospital Anxiety and Depression Scale (Zigmond & Snaith, 1983); Insomnia Severity Index (Bastien, Vallieres, & Morin, 2001); Cognitive Failure Questionnaire (Broadbent, Cooper, FitzGerald, & Parkes, 1982); Perceived Stress Scale (Levenstein et al., 1993); and Multidimensional Fatigue Inventory (Smets, Garssen, Bonke, & De Haes, 1995). Participants will also answer background questions regarding age, education, gender identity, occupation, time since injury/diagnosis, diagnosis, number of hours slept last night, and medication, including medication during the last 24 hours.

Data Collection and Management

Assessment Procedure

For peer review only - http://bmjopen.bmj.com/site/about/guidelines.xhtml

After reading and signing informed consent forms, patients will be randomized into one of two test sequences – either beginning with performing the Mindmore Remote tests at home, followed by traditional neuropsychological testing on site at the clinic, or vice versa (Figure 1). Mindmore Remote testing will be performed by the participants in their homes after being contacted by the psychologist by phone. The remote session will not be directly observed by the psychologist. In cases where the data collection is a part of a larger neuropsychological examination, the validation battery will be performed first during that examination, to avoid effects of fatigue. The test will be administered in the order outlined in table 1. Healthy controls will be randomized in a similar manner as patients, and except that data collection might be a part of a larger neuropsychological examination for patients, the data collection will not differ between groups.

All data collection will be performed by psychologists trained specifically for the task of neuropsychological examinations. The two test sessions will be performed within two weeks, with at least one day of resting in between. Each test session will last about 30-45 minutes, adding up to 1-1,5 hours of testing time for both assessments in total. As part of the traditional neuropsychological examination, patients will complete self-assessment questionnaires. After data collection, a subsample of 10 patients will be contacted and offered to participate in a semi-structured telephone interview regarding research question 3, covering themes of usability and the difference of undergoing neuropsychological assessment on a computer at home vs at the clinic with a psychologist.

[Insert Figure 1. Flow-chart of the study here]

Sample Size

To be able to show that one patient group has a different cognitive profile compared to other patients or healthy controls, the aim is to include 50 participants in each group, which is the recommendation for sample size in normative studies of neuropsychological tests(24). With alpha = .05 and a power of 80%, 16 participants would be enough to detect differences of one standard deviation from the mean. With the goal of 50 participants in each group, this means a statistical power of 0.999 to detect

For peer review only - http://bmjopen.bmj.com/site/about/guidelines.xhtml

differences of one standard deviation from the mean, and a statistical power of 0.706 to detect differences of 0.5 standard deviations from the mean.

Data Analysis Plan

Validation of Mindmore test against traditional paper-pencil neuropsychological test will be performed using Pearson and/or Spearman correlation coefficients depending on distribution of data. To evaluate bias between methods, the Bland-Altman method(25) will be used. In addition, multiple linear regressions will be performed to investigate and adjust for the influence of background variables, such as age, education, gender, diagnosis, and time since injury/diagnosis. To establish neuropsychological profiles of diagnostic groups, descriptive data from each test will be presented, and analysis of variance, with and without covariates, will be performed to determine differences between groups. Due to multiple statistical analyses, adjustments for multiple comparisons will be considered.

Patient and public involvement

Local patient organizations have been informed about the research project, and results from the study will be presented at meetings of these organizations.

Ethics and Dissemination

The study protocol has been approved by the Swedish Ethical Review Authority (2022-06230-01). Before initiating any form of neuropsychological assessment, all participants will be given oral and written information about the study and their participation and will sign informed consent. All participants are informed that they can abort their participation at any given time, without having to give reasons for this. For patients, participating in the study or not will not affect regular health care given at the clinic.

For peer review only - http://bmjopen.bmj.com/site/about/guidelines.xhtml

Undergoing a neuropsychological examination (remotely or traditionally) does not come with any risks in itself. However, it might be stressful and anxiety-provoking, especially for participants getting the impression that they are not performing well. This is a common clinical procedure, and the test leaders, all clinical psychologists, are used to handle such reactions during test sessions. In the traditional testing, such fears can be identified and counteracted to a greater degree than during remote testing. We therefore collect reports of how a sub-sample experience the testing situations pertaining to possible differences in negative affective states (objective 3)

Data management

After inclusion, all participants will be assigned a code. All study material will be coded, and a code key is stored separately in a secure compartment, only accessible to researchers within the project. Only personnel within the research project will have access to data. Study data will be stored for at least ten years after completion of the study.

For Mindmore Remote, the data storage is sophisticated and secure, and no personal data is stored on Mindmore's servers. The invitation to the test is sent by a pseudonymized link, and only the researchers within the project have access to the code, as stated above. In this way, Mindmore AB cannot identify any person taking the test, although the test results are stored on their server.

Plans for communicating important protocol amendments to relevant parties

In case the study protocol will undergo modifications or amendments, an additional ethical application will be submitted to the Swedish Ethical Review Authority. Accordingly, changes will be made in the trial registry (Clinicaltrials.gov).

Dissemination

Results from the study will be published in peer-reviewed open access scientific journals. Further, results will also be presented at national and international scientific conferences, as well as shared with patient organizations and health care officials and policy makers.

For peer review only - http://bmjopen.bmj.com/site/about/guidelines.xhtml

Authors' contributions

NB is the principal investigator and responsible for the project, and for the reporting of results from the project. Data collection will be performed by clinical neuropsychologists at Neuro-Head-Neck Centre, Umeå University hospital, all under the supervision of the principal investigator. NB wrote the initial draft of the manuscript. LA contributed to the study design and revised the manuscript for intellectual content.

Funding

The study is supported by the Research and Development Fund granted by the County Council of Västerbotten [RV-978915], Stiftelsen Promobilia [A23004], the Swedish Neuropsychological Society [Grant number N/A] and a memorial gift to the County Council of Västerbotten regarding research at Umeå University Hospital [Grant number N/A].

Competing interests

None declared. Neither authors nor personnel involved in the research project are affiliated with Mindmore AB.

Patient consent for publication

Not applicable.

Acknowledgements

The authors would like to thank Malin Nygård, Sofia Näckter, Emma Wärn, Felix Sjögren and Therese Eriksson for assisting in data collection.

For peer review only

References

1. Dikmen SS, Machamer JE, Winn HR, Temkin NR. Neuropsychological Outcome at 1-Year Post Head-Injury. Neuropsychology. 1995;9(1):80-90.
2. van der Naalt J, van Zomeren AH, Sluiter WJ, Minderhoud JM. One year outcome in mild to moderate head injury: the predictive value of acute injury characteristics related to complaints and return to work. J Neurol Neurosurg Psychiatry. 1999;66(2):207-13.
3. Sun JH, Tan L, Yu JT. Post-stroke cognitive impairment: epidemiology, mechanisms and management. Ann Transl Med. 2014;2(8):80.
4. Yang Y, Tang BS, Guo JF. Parkinson's Disease and Cognitive Impairment. Parkinsons Dis. 2016;2016:6734678.
5. Benedict RHB, Amato MP, DeLuca J, Geurts JJG. Cognitive impairment in multiple sclerosis: clinical management, MRI, and therapeutic avenues. Lancet Neurol. 2020;19(10):860-71.
6. Holmes GL. Cognitive impairment in epilepsy: the role of network abnormalities. Epileptic Disord. 2015;17(2):101-16.
7. Pendergrass JC, Targum SD, Harrison JE. Cognitive Impairment Associated with Cancer: A Brief Review. Innov Clin Neurosci. 2018;15(1-2):36-44.
8. Lezak MD, Howieson DB, Loring DW. Neuropsychological Assessment. 4 ed. New York: Oxford University Press; 2004.
9. Buckley RF, Sparks KP, Papp KV, Dekhtyar M, Martin C, Burnham S, et al. Computerized Cognitive Testing for Use in Clinical Trials: A Comparison of the NIH Toolbox and Cogstate C3 Batteries. J Prev Alzheimers Dis. 2017;4(1):3-11.
10. Germine L, Reinecke K, Chaytor NS. Digital neuropsychology: Challenges and opportunities at the intersection of science and software. Clin Neuropsychol. 2019;33(2):271-86.
11. Bjorngrim S, van den Hurk W, Betancort M, Machado A, Lindau M. Comparing Traditional and Digitized Cognitive Tests Used in Standard Clinical Evaluation - A Study of the Digital Application Minnemera. Front Psychol. 2019;10:2327.
12. Daniel MH, Wahlstrom D, Zhang O. Equivalence of Q-interactive® and Paper Administrations of Cognitive Tasks: WISC®–V: NCS Pearson Inc.; 2014.
13. Van Patten R. Introduction to the Special Issue - Neuropsychology from a distance: Psychometric properties and clinical utility of remote neurocognitive tests. J Clin Exp Neuropsychol. 2021;43(8):767-73.
14. Delis DC, Kaplan E, Kramer JH. D-KEFS - Delis Kaplan Executive Functions System – Technical Manual. San Antonio: Pearson; 2001.
15. Wechsler D. Wechsler Memory Scale - Third Edition. San Antonio: Psychological Corporation; 1997.
16. Wechsler D. Wechsler Adult Intelligence Scale—Fourth Edition. San Antonio: Pearson; 2008.
17. Brearly TW, Shura RD, Martindale SL, Lazowski RA, Luxton DD, Shenal BV, et al. Neuropsychological Test Administration by Videoconference: A Systematic Review and Meta-Analysis. Neuropsychol Rev. 2017;27(2):174-86.
18. Marra DE, Hamlet KM, Bauer RM, Bowers D. Validity of teleneuropsychology for older adults in response to COVID-19: A systematic and critical review. Clin Neuropsychol. 2020;34(7-8):1411-52.
19. Singh S, Strong RW, Jung L, Li FH, Grinspoon L, Scheuer LS, et al. The TestMyBrain Digital Neuropsychology Toolkit: Development and Psychometric Characteristics. J Clin Exp Neuropsychol. 2021;43(8):786-95.
20. Vermeent S, Spaltman M, van Elswijk G, Miller JB, Schmand B. Philips IntelliSpace Cognition digital test battery: Equivalence and measurement invariance compared to traditional analog test versions. Clin Neuropsychol. 2022;36(8):2278-99.
21. Paterson TSE, Sivajohan B, Gardner S, Binns MA, Stokes KA, Freedman M, et al. Accuracy of a Self-Administered Online Cognitive Assessment in Detecting Amnestic Mild Cognitive Impairment. J Gerontol B Psychol Sci Soc Sci. 2022;77(2):341-50.
22. Van den Hurk W, Bergman I, Machado A, Bjermo J, Gustavsson A. Swedish Normative Data for Mindmore: A Comprehensive Cognitive Screening Battery, Both Digital and Self-Administrated. J Int Neuropsych Soc. 2022;28(2):188-202.

For peer review only

For peer review only - http://bmjopen.bmj.com/site/about/guidelines.xhtml

23. von Elm E, Altman DG, Egger M, Pocock SJ, Gøtzsche PC, Vandenbroucke JP. The Strengthening the Reporting of Observational Studies in Epidemiology (STROBE) statement: guidelines for reporting observational studies. Ann Intern Med. 2007;147(8):573-7.
24. Mitrushina MN, Boone KB, D'Elia LF. Handbook of Normative Data for Neuropsychological Assessment. New York: Oxford University Press; 1999.
25. Giavarina D. Understanding Bland Altman analysis. Biochem Med (Zagreb). 2015;25(2):141-51.

For peer review only

For peer review only - http://bmjopen.bmj.com/site/about/guidelines.xhtml

Tables

Table 1: Outcome Measures

Mindmore Remote Test	Description	Main Outcomes	Validity Test
Symbol Digit Processing Test	SDPT is a test of attention and mental processing speed. The participant views a digit-symbol code key on the top of the screen. In the middle of the screen, one symbol appears. The task is to press the correct number as quickly as possible using the mouse on a number pad at the bottom of the screen.	Number of correct items coded over 90 seconds.	Coding from WAIS-IV (Wechsler, 2010)
Rey Auditory Verbal Learning Test	RAVLT is a test of verbal learning and episodic memory. The participant will hear 15 words presented over five trials, with a recall task after each trial. After the learning trial, a distraction list of 15 words is administered, and after this the task is to recall the first list once again. After 30 minutes (after Stroop below), a free recall retention trial is performed, and after this, a recognition trial, where the participant is supposed to identify the words of the first list in a list of 15 hits and 15 distractors. All responses are given verbally.	Total Learning - Number of words recalled during all five trials;<br>Short term Recall - Number of words recalled after the distraction list;<br>Long term recall - Number of words recalled after 30 minutes;<br>Recognition - Number of correct hits and true negatives during recognition	Word List Recall I and II from WMS-III (Wechsler, 1997)
Corsi Block	Corsi Block is a test of short-term and working memory. On the screen, ten white squares will appear. These will blink in yellow in specific sequences of increasing length, starting with two items. Two different sequences will be presented of each length. After each sequence has finished, the participant is supposed to click with the mouse on the squares in the same order (Corsi Forwards), or in reverse order (Corsi Backwards). If the participant fails to produce the correct sequence in both trials on the same level, the test will end. Corsi Forwards is performed first, and then Corsi Backwards.	Corsi Block Forwards - Number of correct trials in the forward condition; Corsi Block Backwards - Number of correct trials in the Backward condition;	Block Span from WMS-III (Wechsler, 1997)

For peer review only

Trail Making Test - Click	TMT is a test of attention, visuomotor speed and the executive function of switching. The test consists of three conditions. In condition 1 (Number Sequencing), the participant will see numbers 1 to 25 in small circles on the screen and is supposed to click on the numbers in the correct order. In condition 2 (Switching), both numbers 1-13 and letters A-M appears in the circles. The participant is supposed to click on the letters and numbers in order, but each number and letter interchangeably - 1-A-2-B-3-etc. In condition 3 (Motor Speed), the circles are connected by a dotted line, and the participant is instructed to click on the circle in the same order as the line connects them.	Time to completion of each trial	Trail Making Test from D-KEFS (Delis et al., 2001)
Stroop	Stroop is a test of the executive function inhibition, performed in two conditions. In condition 1 (Color Naming and Reading) a color word appear, printed in the same color. The participant is instructed to click as quickly as possible on the correct color word (blue, red, green or yellow), spelled out in black color below the target word. In condition 2 (Inhibition) the color words are written in a different color. The participant is instructed to click, as quickly as possible, on the color word written in black color below the target word that matches the color of the word, and not the word in itself.	Time to completion of each trial	Color-Word Interference Test from D-KEFS (Delis et al., 2001)
FAS	In FAS, a test of verbal production and executive functions, the participant is instructed to say as many words as possible during one minute, starting with a specific letter. The test is performed in three trials using letters F, A, and S, respectively.	Number of words produced over all trials.	Verbal Fluency from D-KEFS (Delis et al., 2001)

Note: WAIS = Wechsler Adult Intelligence Scale; WMS = Wechsler Memory Scale; D-KEFS = Delis-Kaplan Executive Functions System. All tests will be performed using Swedish versions.

293

For peer review only

For peer review only - http://bmjopen.bmj.com/site/about/guidelines.xhtml

Figure caption

Figure 1. Flow-chart of the study.

Note: After signing informed consent (1), participants will be randomized (2) into performning traditional neuropsychological examination (NPS) at the clinic first and then Mindmore remote at home or vice versa (3).

For peer review only

For peer review only - http://bmjopen.bmj.com/site/about/guidelines.xhtml

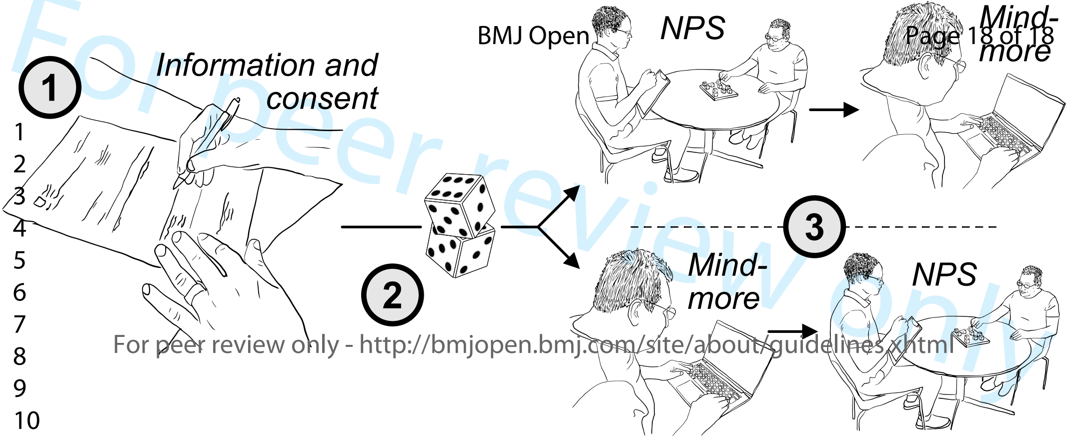

For peer review only - http://bmjopen.bmj.com/site/about/guidelines.xhtml
